# Failed sperm retrieval from severely oligospermic or non-obstructive azoospermic patients on oocyte retrieval day: Emergent oocyte cryopreservation is a feasible strategy

**DOI:** 10.1371/journal.pone.0224919

**Published:** 2019-11-18

**Authors:** Pin-Yao Lin, Chun-Chia Huang, Hsiu-Hui Chen, Bo-Xuan Huang, Maw-Sheng Lee

**Affiliations:** 1 Division of Infertility, Lee Women's Hospital, Taichung, Taiwan; 2 Institute of Medicine, Chung Shan Medical University, Taichung, Taiwan; 3 Department of Urology, Puli Christian Hospital, Nantou, Taiwan; 4 Department of Obstetrics and Gynecology, Chung Shan Medical University Hospital, Taichung, Taiwan; Peking University Third Hospital, CHINA

## Abstract

**Purpose:**

Unexpected sperm retrieval failure on the day of oocyte retrieval is not common but frequently happened in patients with severe oligospermia or non-obstructive azoospermia(NOA). Oocyte cryopreservation is a common strategy after failed collection of sperm when concurrent ovarian stimulation is underwent. However, the use of oocyte vitrification in such male-infertility cases remains unclear.

**Objective:**

To investigate the outcomes of emergent oocyte cryopreservation after failed sperm retrieval from severe oligospermic or non-obstructive azoospermic (NOA) patients on oocyte retrieval day.

**Methods:**

Design: Retrospective cohort study

Setting: Academic fertility center at Lee Women's Hospital, Taiwan, between March 2015 and August 2017.

Patients: For 203 couples with NOA(n = 200) or severe oligospermia(n = 3), testicular spermatozoa (n = 67 cycles) or frozen donor sperm (n = 209 cycles) were injected into fresh or frozen-thawed oocytes via 276 intracytoplasmic sperm injection (ICSI) cycles.

Main Outcome Measures: Clinical pregnancy and live-birth rates (LBRs).

**Results:**

In the 67 cycles involving the use of fresh testicular spermatozoa, no significant differences were observed between fresh and warmed oocytes with respect to the fertilization rates (69.2% vs. 74.1%; p = 0.27), number of Day-3 embryos (8.6±4.4 vs. 6.4±3.4; p = 0.08), number of good-quality Day-3 embryos (4.5±3.9vs. 4.7±3.0; p = 0.45), implantation rates (29.1% vs. 17.8%; p = 0.21), clinical pregnancy rates (36.4% vs. 26.8.0%; p = 0.81), live birth rates (36.4% vs. 14.3%; p = 0.46), or perinatal outcomes. In the 209 cycles involving the use of frozen donor sperm, no significant differences were seen between the two groups, except that the mean birth weights were significantly lower with fresh oocyte pregnancies than with warmed oocytes (2952±196 gm vs 2643±700 gm; p = 0.006).

**Conclusions:**

Emergent oocyte cryopreservation is a feasible strategy to manage unexpected sperm retrieval failure from severe oligospermic or NOA patients on the oocyte retrieval day. There is no detrimental effect on the live birth rate when testicular spermatozoa or frozen donor sperm are injected into the thawed oocytes compared with fresh oocytes.

## Introduction

Unexpected sperm retrieval failure on the day of oocyte retrieval is not common but frequently happens in patients with severe oligospermia or non-obstructive azoospermia (NOA). Oocyte cryopreservation is a common strategy after failed collection of sperm when concurrent ovarian stimulation is underwent. Although concurrent ovarian stimulation for the situation is still on debate with controversies, oocyte-freezing is the only way if ovarian stimulation has been underwent. However, the use of oocyte vitrification in such male-infertility cases remains unclear. There are few reports on the outcomes of subsequent use of testicular spermatozoa via microdissection testicular sperm extraction (microTESE) or frozen donor sperm on cryopreserved oocytes.

Published data on oocyte vitrification/warming has demonstrated acceptable success rates in young, highly selected populations [1–3]. Kushnir et al.[4] indicated that fresh oocytes still represent standard of care due to higher live birth rates. Moreover, data on the feasibility of using frozen oocytes for male-factor infertility is lacking. Compared with fresh oocytes, the main concern is the decrease in the success rate when using frozen eggs for severe male infertility. Therefore, we conducted a retrospective study to examine the clinical outcomes of emergent oocyte cryopreservation after failed sperm retrieval from severely oligospermic or NOA patients on oocyte retrieval day.

## Methods

### Patient selection

This retrospective observational, single-center cohort study included 203 patients who underwent 276 intracytoplasmic sperm injection (ICSI) cycles with fresh or frozen-thawed oocytes for couples with NOA or severe oligospermia at the Lee Women's Hospital, Taiwan between March 2015 and August 2017. Azoospermia was diagnosed when the absence of sperm was observed in two semen samples, in accordance with the World Health Organization guidelines. [5]. All patients had a clinical work-up with physical examination, endocrine profile test (follicle stimulating hormone (FSH), luteinizing hormone (LH) and testosterone) and genetic analysis [6, 7]. Scrotal and transrectal ultrasounds [8] were performed on indication. Exclusion criteria for testicular biopsy were (1) previous testicular-sperm retrieval failure ≥ 2 times; (2) patients with a known abnormal karyotype and Yq deletions; (3) previous testicular sperm from microTESE was highly pathological coupled with poor fertilization and poor grading of embryos. If NOA patients were not eligible for testicular biopsy, transferring to sperm donation program was arranged for them directly. Donor-semen was originated from one specific heathy adult after matching without consanguinity according to country’s legislation.

Frozen donor sperm was cleared for use after a sexually transmitted disease screening 6 months after donation, in accordance with Taiwanese law.

The NOA male partners underwent microTESE[5] on the oocyte retrieval day. If spermatozoa were available with microTESE, ICSI was performed after oocyte pick-up (OPU) 3 hours later.[9] If surgical sperm retrieval failed, oocytes were frozen with the vitrification method and then the couple either underwent another microTESE procedure(2 or more attempts depending on clinical condition) or was referred to a sperm donation program, depending on the pathology of the testicular biopsy. For male partners with severe oligospermia, oocytes were also frozen if unexpected azoospermia occurred on oocyte retrieval day and surgical sperm retrieval was performed if still azoospermic. The retrospective data analysis was approved by the Institutional Review Board of Chung Shan Medical University, Taichung, Taiwan (CS-09054) and all patients provided written informed consent before their microTESE biopsies.

### MicroTESE procedure

The procedures were performed under local anesthesia and started on the testicle with larger volume.[10] The scrotum was incised longitudinally for 3 cm on the median raphe and the testis was then delivered through the incision. MicroTESE was performed using an operative microscope (Carl Zeiss, OPMI Surgical Microscope, Germany) to expose the seminiferous tubules. The testicle was then split open bluntly and tubules were retrieved with jewelers’ forceps from different sites of the two testicular sections. A fragment of testicle was washed in 1 ml mHTF [11] with 5% Serum Substitute Supplement (SSS, Irvine Scientific, Santa Ana, CA, USA), fixed in Bouin’s solution (1 ml) for pathology. The surgeon extracted the tissue fragments (TESE sample) and placed it in a Petri dish. At the end of the procedure, the albuginea incision was closed with a VICRYL 6/0 running suture. The embryologist opened up the seminiferous tubules by mechanically dissecting the tissue with glass cover slides before sperm search. The TESE sample was then centrifuged (600G for 10 min) and the embryologist continued the sperm search on the more concentrated sample. If sperm was not found on the day of surgery, the oocytes were vitrified.

### In vitro fertilization/Intracytoplasmic sperm injection protocol

Controlled ovarian stimulation, oocyte collection, and denudation were performed as previously described [12]. All patients were administered leuprolide acetate (Lupron, Takeda Chemical Industries, Ltd., Osaka, Japan), started during the mid-luteal phase for downregulation. All patients subsequently received recombinant follicle stimulating hormone (Gona-F; Serono, Bari, Italy) for ovarian stimulation from Day 3 until the dominant follicle reached a diameter of >18 mm, followed by injection of 250 μg human chorionic gonadotropin (Ovidrel, Serono) 36 hours before oocyte retrieval. The retrieved oocytes were cultured in Quinn's Advantage Fertilization Medium (Sage Bio-Pharma, Inc., Trumbull, CT, USA) with 15% serum protein substitute (SPS, Sage BioPharma, Inc.) in a triple gas phase of 5% CO_2_, 5% O_2_, and 90% N_2_. The cumulus cells were removed by pipetting the oocytes in modified human tubal fluid media (mHTF) containing 80 IU/mL hyaluronidase (Type 8, H-3757; Sigma Chemical, St. Louis, MO, USA). Following ICSI, all embryos were further cultured in microdrops of Quinn’s Advantage Cleavage (SAGE) medium [13]. A droplet of medium with prepared spermatozoa was mixed with up to 3.5% polyvinylpyrrolidone (PVP, SAGE Media, USA). Metaphase-II oocytes were placed in an HTF droplet and injected with one spermatozoon each after gentle aspiration of the cytoplasm (modified from Moilanen et al., 1999)[14]. After ICSI,embryonic development, including the embryonic pronuclei appearance (18–20 h) and eight-cell stage (69–70 hours), was observed. The embryo transfer was performed on Day 3 after oocyte retrieval or thawing of oocytes. Day 3 embryos with ≥8 equal-sized blastomeres and ≤20% fragmentation was considered good-quality embryos.

### Oocytes vitrification/Thawing method

Oocyte vitrification occurred 1–2 hours after cumulus removal. All the oocytes were vitrified and warmed using the Cryotop (Kitazato Co., Fujinomiya, Japan) and Cryotech Vitrification Method (Cryotec, Cryotech, Tokyo, Japan)[12, 15, 16]. Briefly, oocytes were equilibrated at room temperature in the solution supplied with the kit containing ethylene glycol (EG) and dimethylsulphoxide (Me_2_SO) for 15 min. Then the oocytes were moved to a vitrification solution containing a double concentration of EG + Me_2_SO, and sucrose or trehalose for 60–90s. Oocytes were then loaded on the top of the film strip supplied with the kit, and the sample was quickly immersed into liquid nitrogen for storage. At warming, the strip was immersed directly into the kit warming solution at 37°C for 1 min and then oocytes were incubated once for 3 min and twice for 5 min in the kits’ diluent and washing solutions, respectively. After warming, oocytes were cultured in HTF medium for 2–3 h and then used for ICSI. Embryo transfer was performed on day 3 after oocyte retrieval or thawing oocytes. In fresh oocytes group, luteal phase support was started after egg-retrieval with Crinone (1.125 g, 8% gel; vaginal suppositories, Merck Serono, UK) application daily and oral dydrogesterone (10 mg; Duphaston, Abbott Biologicals B.V., the Netherlands) three times a day for 14 days. In thawing oocytes group, patients underwent an artificial cycle for endometrial preparation with hormone replacement as previously published.[12]

Clinical pregnancy was defined as the presence of an intrauterine gestational sac with positive cardiac movement on ultrasound at 6–8 weeks [17]. Pregnancy outcome was tracked for all pregnant women by mailed questionnaire or by phone. The live birth rate (LBR) was defined as the proportion of IVF cycles reaching embryo transfer that resulted in the birth of at least one live-born child. Cumulative pregnancy rate was followed up till May 2018.

### Statistical analysis

The primary outcome measure was the live birth rate between the groups with fresh oocytes and warmed oocytes. No formal sample size calculation was performed but all available patients in our center were included in the study.

Quantitative variables, representing differences between groups of patients in mean±SD, and categorical variables, representing differences in distributions, were tested with the Kruskal-Wallis one-way analysis of variance (ANOVA) and the Chi-square test, respectively. Non-normally distributed continuous variables are expressed as medians (interquartile range) unless otherwise stated. Continuous and categorical variables were compared between groups using the Kruskal-Wallis test and Fisher’s exact test, respectively. The statistical analysis was conducted using the Statistical Program for Social Sciences (SPSS Inc., Version 15.0, Chicago, U.S.A.) and MedCalc Statistical Software version 16.8 (MedCalc Software, Ostend, Belgium; 2016). All p-values were two-sided, and values less than 0.05 were considered statistically significant.

## Results

### Clinical outcome of fresh testicular spermatozoa

During the study period, 276 fresh ICSI cycles from 203 couples were included in the retrospective study. A total of 67 cycles used fresh testicular spermatozoa to inseminate the fresh or warmed oocytes. 11 cycles (3 cycles from severely oligospermic patients and 8 cycles from NOA patients) used frozen eggs because of previous sperm-retrieval failure with unexpected egg-freezing at that time.

Table 1 illustrates the baseline characteristics and clinical outcome of using fresh testicular spermatozoa ICSI cycles. The sperm retrieval rate of microTESE for NOA patients was 45.7%. The post-thawing survival rate of warmed eggs was 87.2% and 3 cases were canceled in the warmed egg group due to no embryo to transfer (cancellation rate: 27%). No significant differences between the groups of fresh oocytes and warmed oocytes were observed in fertilization rates (69.2% vs. 74.1%; p = 0.27), number of Day-3 embryos (8.6±4.4 vs. 6.4±3.4; p = 0.08), number of good-quality Day-3 embryos (4.5±3.9 vs. 4.7±3.0; p = 0.45), implantation rates (29.1% vs. 17.8%%; p = 0.21),clinical pregnancy rates (36.4% vs. 26.8.0%; p = 0.81), or live birth rates (36.4% vs. 14.3%; p = 0.46). Obstetric outcomes indicated 4 pregnancies with 6 healthy infants born (4 singletons and 1 set of twins) in the warmed-oocyte group and 8 pregnancies with 10 healthy infants born (6 singletons and 2 sets of twins) in the fresh-oocyte group. All 15 infants had normal karyotypes. No differences in perinatal outcomes were noted between the two groups.

**Table 1:** Characteristics and outcomes of ICSI cycles using fresh testicular spermatozoa in fresh oocytes or warmed oocytes.

	Warmed oocytes	Fresh oocytes	p value
**No. of cycles**	11	56	
**No. of patients**	10	50	
**Age of male partners (y)**	38.9±3.9	39.1±6.7	0.92
** • Serum FSH (mIU/ml)**	15.9(10.8–2.1)	18.6(15.9–26.4)	0.75
** • Serum LH (mIU/ml)**	5.1(3.6–6.6)	7.5(6.1–8.9)	0.81
** • Serum testoterone(ng/ml)**	1.28(0.65–1.91)	1.7(0.61–2.81)	0.87
**Age of female partners (y)**	33.8±3.1	35.6±3.9	0.16
**Day3 serum FSH (mIU/ml)**	5.53±2.3	6.2±2.3	0.38
**Duration of infertility (y)**	4.6±3.1	3.57±3.3	0.37
**No. of oocytes retrieved**	16.4±10	12.1±7.5	0.11
**No. of mature oocytes retrieved**	13±5.4	10.2±5.4	0.24
**Fertilization rate, % (n)**	69.2% (90/130)	74.1% (403/544)	0.27
**No. of total Day 3 embryos**	8.6±4.4	6.4±3.4	0.08
**No. of good-quality Day 3 embryos**	4.5±3.9	4.7±3.0	0.45
**No. of embryos transferred**	2.2±1.5	1.8±1.6	0.48
**Implantation rate, % (n)**	29.1% (7/24)	17.8% (18/101)	0.21
**Clinical pregnancy rate, % (n)**	36.4% (4/11)	26.8% (15/56)	0.81
**Live birth rate, % (n)**	36.4% (4/11)	14.3% (8/56)	0.46
**Abortion rate, % (n)**	0% (0/4)	30% (5/15)	0.18
**Multiple gestation, % (n)**	25% (1/4)	25% (2/8)	1
**Gestational age (weeks)**	37.7±0.5	37.7±1.2	1
**Birth weight (gm)**	3037±315	2881±373	0.49

Note: Values are % (n) or mean± SD or medians (interquartile range); P <0.05

### Clinical outcomes of frozen-thawed donor sperm

A total of 209 ICSI cycles used frozen-thawed donor sperm to inseminate fresh or warmed oocytes. 15 cycles used frozen oocytes from NOA because of previous TESE failure with unexpected egg-freezing at that time. Overall, the post-thawing survival rate of warmed eggs was 86.9% and 1 case was cancelled in the warmed egg group due to no embryo for transfer (cancellation rate: 6.7%). The baseline characteristics, controlled ovarian hyperstimulation (COH) cycle parameters, and clinical outcomes of donor sperm ICSI cycles are presented in Table 2.

**Table 2:** Characteristics and outcomes of ICSI cycles using frozen-thawed donor sperm in fresh oocytes or warmed oocytes.

	Warmed oocytes	Fresh oocytes	p value
No. of cycles	15	194	
No. of patients	14	129	
Age of male partners (y)	38.9±4.9	39.8±6.3	0.91
Serum FSH (mIU/ml)	16.7(7.8–25.6)	25.6(9.8–29.2)	0.65
Serum LH(mIU/ml)	8.5(6.9–10.1)	19.5(9.8–29.2)	0.12
Serum testoterone(ng/ml)	1.8(0.57–3.0)	1.71(0.6–2.8)	0.87
Age of female partners (y)	30.93±4.0	32.5±3.6	0.11
Baseline FSH (mIU/ml)	6.71±4.1	6.83±4.1	0.23
Duration of infertility (y)	3.14±2.7	3.96±2.98	0.32
No. of oocytes retrieved	17.3±8.7	12.9±7.4	0.11
No. of mature oocytes retrieved	14±5.6	10.2±4.6	0.38
Fertilization rate, % (n)	67.9% (133/196)	71.7% (1522/212)	0.26
No. of total Day 3 embryos	9.21±5.1	6.71±5.5	0.32
No. of good-quality Day 3 embryos	6.5±4.7	4.7±3.2	0.35
No. of embryos transferred	3.2±0.6	2.8±0.8	0.29
Implantation rate, % (n)	28.9% (13/45)	30.9% (176/568)	1
Clinical pregnancy rate, % (n)	66.7% (10/15)	51.0% (99/194)	0.29
Abortion rate, % (n)	20% (2/10)	18.2% (18/99)	1
Live birth rate, % (n)	53.3% (8/15)	41.7% (81/194)	0.298
Multiple gestation, % (n)	25% (2/8)	45.7% (37/81)	0.25
Gestational age (weeks)	38.2±1.2	36.5±2.96	0.119
Birth weight (gm)	2952±196	2643±700	0.006^a^

Note: Values are % (n) or mean± SD or medians (interquartile range)

^a^P<0.05

Fertilization rates (67.9% vs. 71.7%; p = 0.26), number of Day-3 embryos (9.21±5.1 vs. 6.71±5.5; p = 0.32), number of good-quality Day-3 embryos (6.5±4.7 vs. 4.7±3.2; p = 0.35), and implantation rates (28.9% vs. 30.9%; p = 1.0) were similar between the groups of fresh oocytes and warmed oocytes. No significant differences were observed between the two groups in clinical pregnancy rate (66.7% vs. 51.0%; p = 0.29), abortion rate (20% vs. 16.2%; p = 0.52), or live birth rates (53.3%vs. 42.7%; p = 0.30). Despite no differences with respect to gestational age at delivery, the mean birth weights were significantly lower with fresh oocyte pregnancies, both in singletons and twins, than with warmed oocytes (2952±196 gm vs. 2643±700 gm; p = 0.006).

## Discussion

Effective oocyte cryopreservation improves and extends the current assisted reproductive technologies [18];however, Kushnir et al. recently reported that fresh oocytes still represent a higher standard of care due to higher live birth rates [4]. There is little data on the feasibility of using frozen oocytes in male-factor infertility. This report aimed to compare the clinical outcomes of fresh and warmed oocytes via ICSI with testicular spermatozoa or frozen donor sperm from severely oligospermic or NOA patients. Our primary findings were: (1) The pregnancy outcomes between fresh and frozen oocytes were comparable when ICSI was performed using fresh testicular spermatozoa and (2) If surgical retrieval of spermatozoa failed, the outcomes of fresh and frozen oocytes when using frozen donor sperm were similar.

NOA is reported in about 60% of azoospermic patients and 15% of all infertile men [19]. MicroTESE appears to improve the frequency of successful sperm retrieval and is suggested to be the standard of the management for NOA [19–25]. However, because spermatogenesis is limited in NOA patients, surgical sperm-retrieval rates are between 36%-64% [19, 26]. Although several micro-surgical methods can improve surgical sperm retrieval rates [27–30], failure of spermatozoa retrieval is still possible. Emergent oocyte cryopreservation is the alternative option if concurrent ovarian stimulation is performed. However, few studies have reported the efficacy of emergent oocyte cryopreservation after failed retrieval of spermatozoa. Our study reported similar outcomes between fresh and frozen oocytes when performing ICSI using surgically retrieved fresh spermatozoa. Frozen oocytes were also competent to be fertilized with testicular spermatozoa and progress to healthy embryos. Using the surgically retrieved fresh or frozen spermatozoa for ICSI in NOA patients is still controversial, with contrasting results. The advantages of frozen testicular spermatozoa are the independent scheduling of sperm and oocyte retrieval, avoidance of unnecessary ovarian stimulation of the female partner (if no sperm is retrieved) and concomitant lessening of stress to the couple. Although most relevant studies [31–36] suggest that fertilization rates using cryopreserved-thawed testicular spermatozoa are similar to those of fresh sperm, the freezing-thawing process does have limitations, primarily loss of spermatozoa or recovery only of non-motile spermatozoa that are ineligible for injection after thawing [37–39]. Immature sperm from testicular tissues are much more sensitive to cryodamage than ejaculated sperm, possibly leading to a higher rate of DNA fragmentation or ruptured plasma membranes [40–43]. Thawed testicular spermatozoa also make sperm selection more difficult due to loss of motility and viability. Therefore, the use of fresh testicular sperm from NOA patients for ICSI is preferred in our center. The findings of An et al. [44] agree with ours that vitrified oocytes combined with fresh spermatozoa, via microTESE, showed similar clinical efficacy compared to fresh oocytes. Instead of freezing vulnerable testicular spermatozoa, cryopreservation of oocytes may result in better clinical outcomes and also makes it possible to use frozen donor sperm in case spermatozoa cannot be retrieved. The only difference noted in our study between frozen and thawed oocytes using frozen donor sperm was significantly higher birth weight, a difference also reported by Levi Setti et al. [45]. This may be due to trends towards higher multiple gestation rate (MGR) and earlier gestational age at delivery in the fresh oocyte group. Further investigation is required. For NOA patients, repeated testicular biopsies may cause irreversible damage like testicular failure. If sperm retrieval is successful via TESE, we preferred to collect more embryos as possible in the same cycle to prevent repeated biopsies. Compared with other IVF patients, lower fertilization rate, less D3 embryos, poorer quality D3 embryos have been observed from NOA couples. The higher multigestation rate (MGR) resulted from more embryos being transfered in this young population. Other possible causes for this high MGR was the selection of male cases with better prognosis and the low number of cases analyzed. In the future and based on our good Day 3 results, proceeding Day 3 ET to blastocyst transfer in NOA couples seems feasible and effective in decreasing MGR. Moreover, time-lapse system for morphokinetics selection [46] and preimplantation genetic testing for aneuploidy (PGT-A) [47] to avoid aneuploid embryos could also assist for embryo selection and decrease MGR.

Unexpected situations can occur during IVF/ICSI treatment, including a male partner who fails to produce semen. Successful live birth after emergency oocyte cryopreservation when sperm extraction failed has been reported [48, 49]. However, the importance of emergent oocyte cryopreservation has not been emphasized for this situation. According to our results, emergent oocyte cryopreservation is an effective strategy to allow for arranging further biopsies and medical treatment or to seek an eligible donor. Segmentation of assisted reproductive technology into ovarian stimulation and sperm retrieval from surgery or donors could have the potential to improve and extend the current assisted reproductive technologies.

The present study has several limitations. First, the study had a small sample size. Thus, a larger sample size is needed to confirm the results of this study. Second, the retrospective study model has a risk of selection bias because the groups originally were assigned for non-random reasons. The male patients who were eligible to receive testicular biopsy may seek donor-sperm because of the concerns on LBR and costs. Therefore, the results of the study may only represent parts of the groups not all due to these limitations. A randomized controlled trial could improve the quality of the findings. Third, only fresh testicular-spermatozoa were used in the study; therefore, the outcomes when using frozen testicular-spermatozoa require further investigation.

## Conclusions

In conclusion, the results indicate that emergent oocyte vitrification is an effective procedure when sperm retrieval fails on oocyte retrieval day. No detrimental effect was seen on the live birth rate when using surgically retrieved spermatozoa or frozen donor sperm to inject the thawed oocytes. Thus, oocyte cryopreservation is a feasible strategy to manage unexpected sperm retrieval failure or azoospermic patients.

## Abbreviations

NOA: non-obstructive azoospermic
ICSI: intracytoplasmic sperm injection
LBRs: live-birth rates
MGR: multiple gestation rate
MicroTESE: microdissection testicular sperm extraction
FSH: follicle stimulating hormone
LH: luteinizing hormone
